# HBV X protein-based therapeutic vaccine accelerates viral antigen clearance by mobilizing monocyte infiltration into the liver in HBV carrier mice

**DOI:** 10.1186/s12929-020-00662-x

**Published:** 2020-05-28

**Authors:** Jau-Hau Horng, Wei-Hsiang Lin, Chang-Ru Wu, You-Yu Lin, Li-Ling Wu, Ding-Shinn Chen, Pei-Jer Chen

**Affiliations:** 1 Graduate Institute of Microbiology, National Taiwan University College of Medicine, No. 1 Jen Ai Road Section 1, Taipei, Taiwan (R.O.C.); 2 TheVax Genetics Vaccine Company Limited, 5F, No. 25, Jen Ai Road Section 4, Taipei, Taiwan (R.O.C.); 3 Graduate Institute of Clinical Medicine, National Taiwan University College of Medicine, No. 1, Jen Ai Road Section 1, Taipei, Taiwan (R.O.C.); 4 Department & Institute of Physiology, National Yang-Ming University, No. 155, Section 2, Linong Street, Taipei, Taiwan (R.O.C.); 5 Division of Gastroenterology, Department of Internal Medicine, National Taiwan University Hospital, No. 1, Changde Street, Taipei, Taiwan (R.O.C.); 6 Hepatitis Research Center, National Taiwan University Hospital, No. 1, Changde Street, Taipei, Taiwan (R.O.C.)

**Keywords:** Chronic hepatitis B, Immunotherapy, Hepatic innate immunity, Myeloid cell

## Abstract

**Background:**

Hepatitis B virus (HBV) persistently infected about 250 million people worldwide, and a curative treatment remains an unmet medical need. Among many approaches to treat chronic hepatitis B (CHB), therapeutic vaccines have been developed for two decades, but none have yielded promising results in clinical trials. Therefore, dissection of HBV clearance mechanisms during therapeutic vaccination in appropriate models, which could give rise to new curative therapies, is urgently needed. Growing evidence indicates that prolonged and intensive exposure of antigen-specific T cells to viral antigens is a major cause of T cell exhaustion, and decreases anti-HBV immunity efficacy of therapeutic vaccination. HBV X protein (HBx) is expressed at low levels, and the understanding of its immunogenicity and potential in therapeutic CHB vaccines is limited.

**Methods:**

HBV genome sequences from CHB patients were cloned into a pAAV plasmid backbone and transfected into immunocompetent mouse hepatocytes through hydrodynamic injection. Mice carrying > 500 IU/mL serum HBV surface antigen (HBs) for more than 4 weeks were considered HBV carriers mimicking human CHB and received 3 doses of weekly HBx vaccine by subcutaneous immunization. Serum HBV clearance was evaluated by monitoring serum HBs and HBV-DNA titers. Residual HBV in the liver was evaluated by western blotting for HBV core antigen. The splenic antigen-specific T cell response was quantified by a 15-mer overlapping peptide-stimulated interferon-γ enzyme-linked immunospot assay. Blood and hepatic immune cells were quantified by flow cytometric analysis.

**Results:**

Our HBx-based vaccine induced systemic HBx-specific CD4^+^ and CD8^+^ T cell responses in HBV carrier mice and demonstrated significant HBs and HBV-DNA elimination. The protective effect persisted for at least 30 days without additional booster immunization. Different infiltrating myeloid cell subsets, each with distinctive roles during immune-mediated HBV clearance, were found in the liver of vaccinated mice. During vaccine therapy, inflammatory monocyte depletion resulted in sustained HBV clearance inhibition, whereas phagocytic monocyte-derived macrophage and Kupffer cell elimination resulted in only transient inhibition of vaccine-induced HBV clearance.

**Conclusions:**

We report the potential role of HBx as a major immunogen in an HBV therapeutic vaccine and the significance of a liver-infiltrating monocyte subset during hepatic viral clearance.

## Background

Despite decades of active vaccination, chronic hepatitis B (CHB) remains highly prevalent, with the World Health Organization estimating there are 250 million carriers worldwide. This results in a heavy disease load of hepatitis B virus (HBV)-related, end-stage liver diseases and a high death toll. At present, there are only two approved treatments for CHB: nucleot(s)ide analogs and pegylated interferon (IFN). Both treatments effectively inhibit viral replication but have limited efficacy in removing persistent viral genomes from infected hepatocytes or in clearing HBV surface antigen (HBs) from the serum. Therefore, exploring new curative therapies and elucidating their mechanisms in appropriate animal models are urgently needed.

T cell exhaustion is commonly observed in CHB patients and is characterized by dysfunctional HBs-, HBV core antigen (HBc)-, and polymerase-specific T cells that express multiple immune inhibitory receptors, such as programmed cell death protein 1 [1–3]. Recent studies have reported this dysfunction to extend to HBs-specific B cells, which also display surface markers of exhaustion and defective antibody production in CHB patients [4]. These adaptive immunity deficiencies in CHB patients are the major cause of the host’s inability to remove HBV-infected hepatocytes and clear HBs from the blood.

Currently, curative immune-therapeutic strategies for CHB, including therapeutic vaccination, immune checkpoint inhibitors, oral Toll-like receptor agonists, and even engineered T cell therapy, are focused on restoring or enhancing host cytotoxic T cell activity to destroy HBV-infected hepatocytes [5]. Several studies have reported promising results, but the approaches require further clinical trials. Among these strategies, therapeutic vaccination is one of the simplest and most straightforward strategies. However, despite more than 20 years of development with some vaccines entering clinical trials, no satisfactory results have been obtained thus far [6]. In one recent clinical trial, the GS-4774 vaccine (a fusion antigen composed of HBs, HBc, and a partial HBV X protein (HBx) sequence) showed modest increases in HBs- and HBc-specific CD8^+^ T cell numbers with an improved cytokine secretion ability in peripheral blood mononuclear cells (PBMCs), and exhausted T cell numbers were also reduced in the total CD8^+^ T cell pool [7]. Nevertheless, the vaccine did not recover sufficient virus-specific CD4^+^ T cells or B cells or enhance innate immune responses and thus failed to reduce the circulating HBs level. Subverting the host’s tolerogenic status to HBV antigens remains the main challenge in developing a curative therapy.

One unique feature of CHB is the excessive levels of viral antigens (HBs, HBc and HBV e antigen) in both infected hepatocytes and the circulation. Such antigens have been proposed as tolerance-inducing antigens [8]. It is well documented in a chronic lymphocytic choriomeningitis virus infection model that persistent high-level viral antigen exposure through somatic cells results in antigen-specific T cell exhaustion [9, 10]. It was also reported that in another liver-specific model, a high antigen expression level in hepatocytes largely suppresses hepatic-primed CD8^+^ T cell function in the liver [11]. Thus, to reduce the HBV antigen load, neutralizing antibodies, RNA interference, and/or nucleic acid polymers have been suggested as combination treatments for HBV immunotherapy [12]. We hypothesize that targeting viral proteins expressed at low levels in hepatocytes has advantages over targeting the abundant ones in restoring the antigen-specific T cell function in the liver. HBx is a prime therapeutic candidate because it controls HBV gene expression and replication but is expressed at low levels during natural infection [13, 14]. While most current HBV therapeutic vaccines usually focus on abundant HBV structural proteins, such as HBs and HBc, as the major immunogens [6], HBx may actually be a more suitable immunogen candidate owing to its low expression levels. Here, we propose using the low-expressed molecule HBx as a therapeutic vaccine antigen as an alternative method to rejuvenate anti-HBV adaptive immunity in CHB. The previously mentioned GS-4774 vaccine failed to increase HBx-specific T cell numbers in CHB patients [7], possibly due to its use of a partial HBx sequence [15]. Therefore, the potential of full-length HBx in therapeutic vaccines cannot be overlooked.

The liver is considered a tolerogenic organ that suppresses T cell functions in an antigen-independent manner and maintains its immunosuppressive environment via multiple mechanisms mediated through resident nonparenchymal cells (NPCs) [16, 17]. This tolerogenic liver environment further heightens the barrier to CHB immunotherapy. Hence, the regulation of liver-resident immunity is another important issue to consider in designing a successful curative therapy. Modulation of the liver-resident immune environment, such as the induction of intrahepatic myeloid cell aggregates for T cell population expansion, facilitates HBV immune clearance in mouse models [18, 19]. The ultimate goal of HBV immunotherapy is to restore sufficient anti-HBV immunity in both the innate and adaptive arms to functionally cure CHB while overcoming T cell exhaustion and the tolerogenic liver environment. In the current study, we propose an HBx-based therapeutic vaccine and evaluate its effect and possible mechanisms with an established immunocompetent HBV-persistent mouse model.

## Methods

### Animals

Male CBA/CaJ or C57BL/6 J mice (6 ~ 8 weeks old) were purchased from the National Laboratory Animal Center (Taipei, Taiwan). Mice were housed in an animal biosafety level 2 facility with < 5 mice per cage in a temperature-controlled room (22 ± 2 °C) with a 12-h light/dark cycle.

### pAAV/HBV1.2 plasmid construct and a hydrodynamic HBV transfection mouse model

A 1.2-fold over-length complete HBV genome sequence isolated from CHB patient serum was cloned into a pAAV plasmid backbone, namely, pAAV/HBV1.2, as described elsewhere [20]. The plasmid DNA was prepared with Endofree Maxiprep kits (QIAGEN, Hilden, Germany). A total of 10 μg of pAAV/HBV1.2 plasmid was dissolved in 8% mouse body weight-equivalent phosphate-buffered saline (PBS) and injected via the mouse tail vein within 5 s. The serum HBs titer was monitored after day 28 post-injection. Animals with a serum HBs titer > 500 IU/mL were considered stable HBV carriers.

### Preparation and administration protocol for the vaccine formulation

RAP1-HBx and RAP1-E7 were constructed by fusing the HBx or human papillomavirus E7 protein (HPV-E7) sequence, respectively, to a Pseudomonas Exotoxin A (PE-A) mimicry sequence to enhance immunogenicity (Supplementary Figure 1) [21]. Both fusion proteins were produced with the *Escherichia coli* expression system (TheVax Genetics Company, Taipei, Taiwan). TVGV-HBx and TVGV-E7 were obtained by mixing 100 μg of RAP1-HBx or RAP1-E7 protein, respectively, with 20 μg of CpG oligodeoxynucleotides (CpG-ODN; TheVax Genetic Company) in 50 μL of PBS. The HPV-E7-containing vaccine served as an antigen specificity control for the HBx-containing vaccine. Vaccine formulations were diluted in PBS if a lower vaccination dose was required. The trace endotoxin level in each vaccine was tested with an endotoxin quantification kit (Lonza, Basel, Switzerland). The total endotoxin quantity per injection was less than 10 EU. Recombinant HBc (rHBc; Xiamen University, Xiamen, China) and thioredoxin-fused recombinant HBx (rHBx; TheVax Genetics Company) were produced with the *Escherichia coli* expression system. The rHBx-based vaccine was used in a comparative experiment with rHBc to prevent the possible bias caused by the immunostimulatory PE-A mimicry sequence. Trace endotoxin in protein preparations was removed with Pierce high-capacity endotoxin removal columns (Thermo Fisher Scientific, Waltham, MA, USA). The rHBc and rHBx vaccine preparation and administration protocols were the same as those described previously.

### Extraction and quantification of serum and liver HBV-DNA

Serum HBV-DNA was extracted with a MagNA Pure LC total nucleic acid isolation kit (Roche, Basel, Switzerland) according to the manufacturer’s protocol. Total liver DNA was extracted by using a Gentra Puregene Tissue kit (QIAGEN). HBV-DNA was quantified by quantitative polymerase chain reaction (Q-PCR) on a LightCycler instrument (Roche). The primer set used for amplification had the sequences 5′-CCGATCCATACTGCGGAAC-3′ and 5′-GCAGAGGTGAAGCGAAGTGCA-3′. The fluorescently labeled hybridization probes had the sequences 5′-LC-Red640-TCTGTGCCTTCTCATCTGCCGGACC-PH-3′ and 5′-TCTTTACGCGGACTCCCC-FLU-3′. Q-PCR was performed with the following conditions: denaturation at 95 °C for 10 min, followed by 45 cycles of denaturation at 95 °C for 3 s, annealing at 53 °C for 10 s, and extension at 72 °C for 16 s. A standard calibration curve was derived with a serially diluted plasmid containing the HBV genotype C sequence.

### Serum viral biomarker analysis

**Fig. 1:**
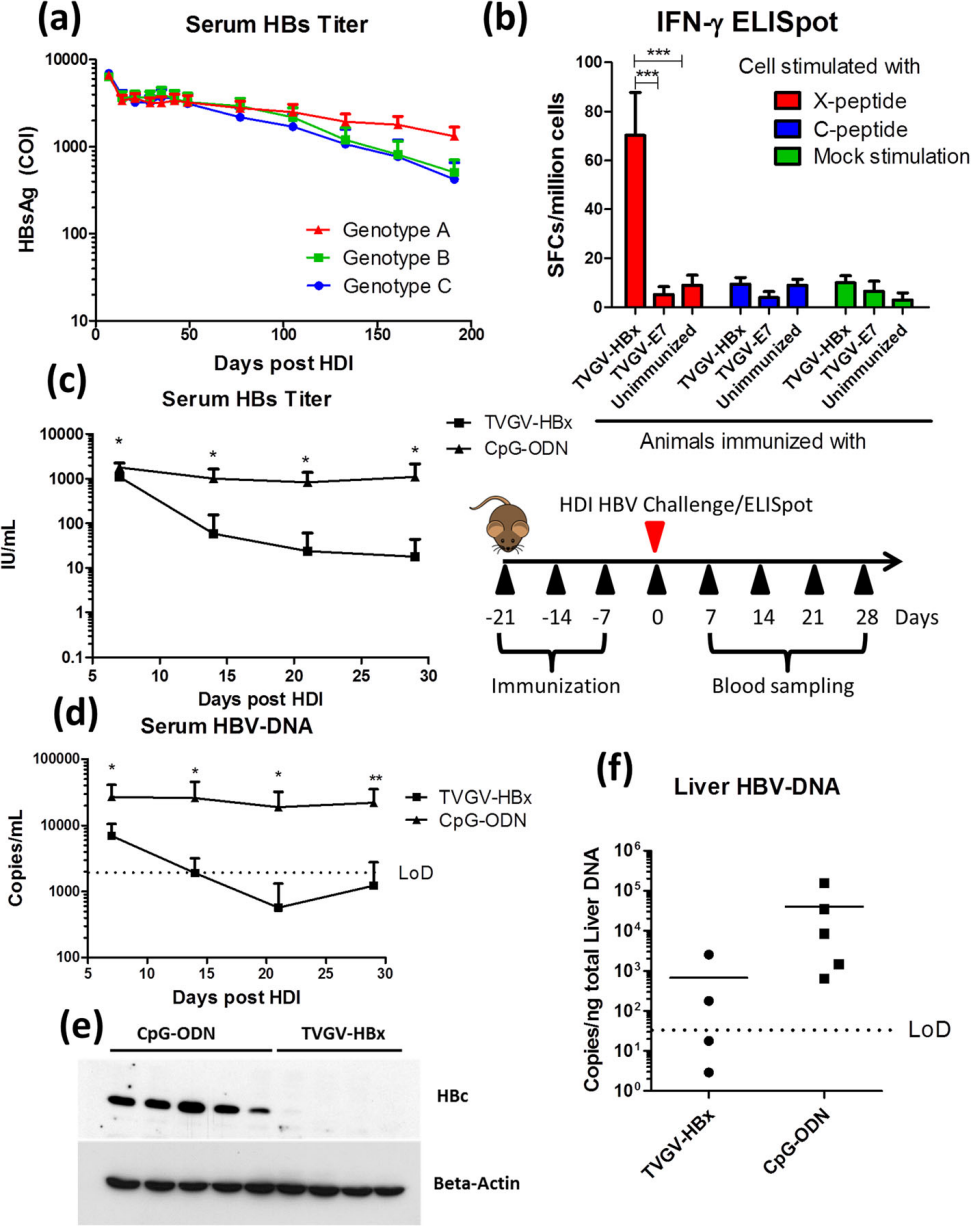
TVGV-HBx exerts a protective function against hydrodynamic HBV exposure in persistence-prone CBA/CaJ mice. **a** Naïve CBA/CaJ mice (N = 9) received hydrodynamic injection of 10 μg of different genotypes of the pAAV/HBV1.2 plasmid on day 0. Blood samples were collected at the indicated time points, and the serum HBs titers are shown. **b** Naïve CBA/CaJ mice (N = 4) received TVGV-HBx or TVGV-E7 or remained untreated on days 0, 7 and 14. Splenocytes were collected on day 21 and subjected to an IFN-γ ELISpot assay. **c**-**f** Naïve CBA/CaJ mice (N = 4 ~ 5) received TVGV-HBx or CpG-ODN immunization every 7 days for 3 consecutive weeks. Mice received HDI transfection with 10 μg of pAAV/HBV1.2 plasmid one week after the last immunization. Mouse serum was sampled on days 7, 14, 21, and 28 after HDI transfection. Liver samples were collected at the endpoint of the experiment. **c** Serum HBs titers were determined. **d** Serum HBV-DNA was detected. **e** Liver HBc was detected by western blotting. **f** Liver HBV-DNA was detected. Statistics: Student’s *t*-test. *, *p* < 0.05; **, *p* < 0.01; ***, *p* < 0.001; COI, cut-off index; LoD, limit of detection; SFCs, spot-forming cells

**Fig. 2:**
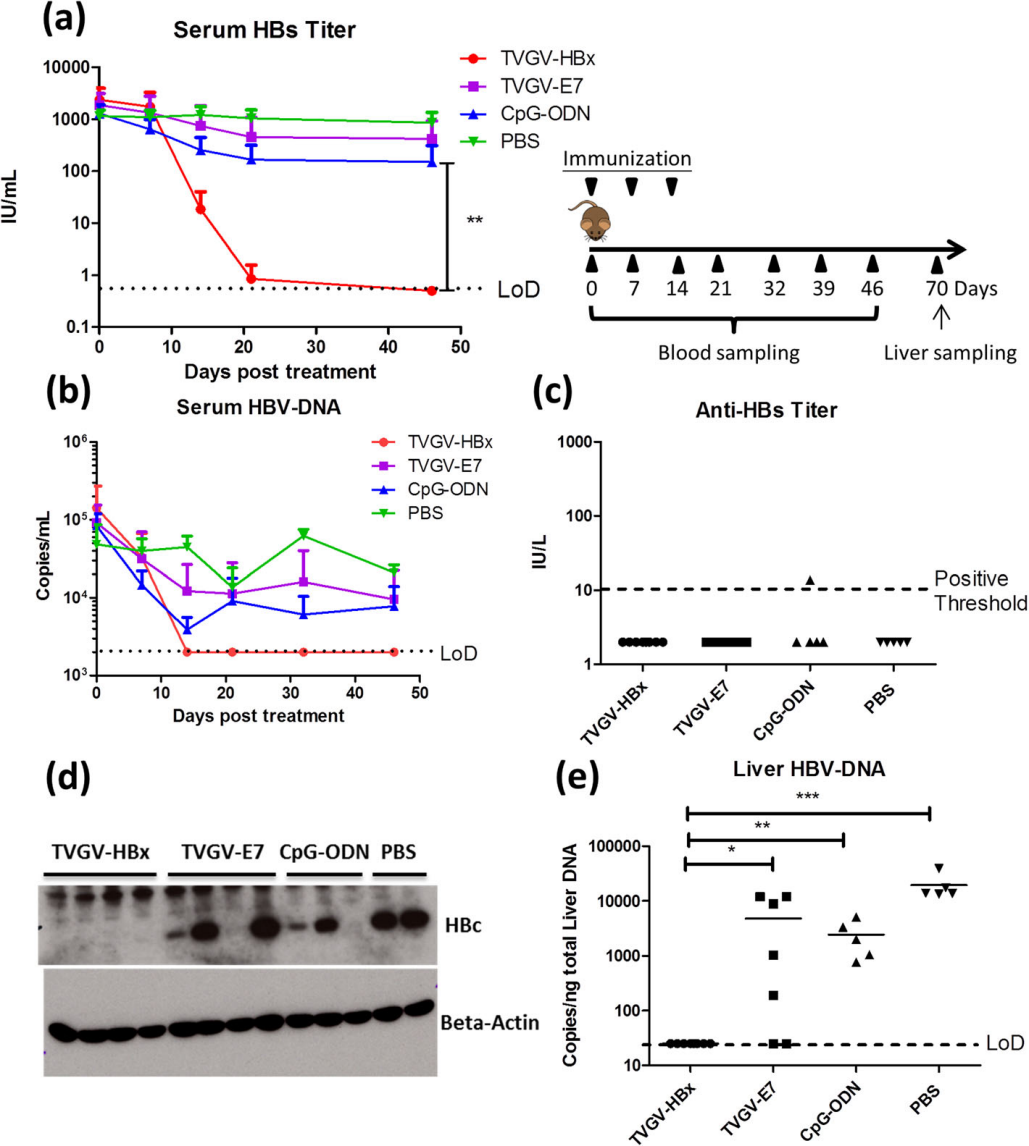
TVGV-HBx exerts therapeutic function in HBV carrier mice. HBV carrier mice (N = 5 ~ 8) received TVGV-HBx, TVGV-E7, CpG-ODN alone or PBS on days 0, 7 and 14. Mouse serum was sampled on days 0, 7, 14, 21, 32, 39 and 46, and liver samples were collected on day 70. **a** Serum HBs titers were determined. **b** Serum HBV-DNA was detected. **c** Serum anti-HBs titer on day 39. **d** Liver HBc was detected by western blotting. **e** Liver HBV-DNA was detected. The value of the undetectable Q-PCR sample was assigned as the detection limit for plotting. Statistics: Student’s *t*-test. *, p < 0.05; **, p < 0.01; ***, p < 0.001; LoD, limit of detection

**Fig. 3:**
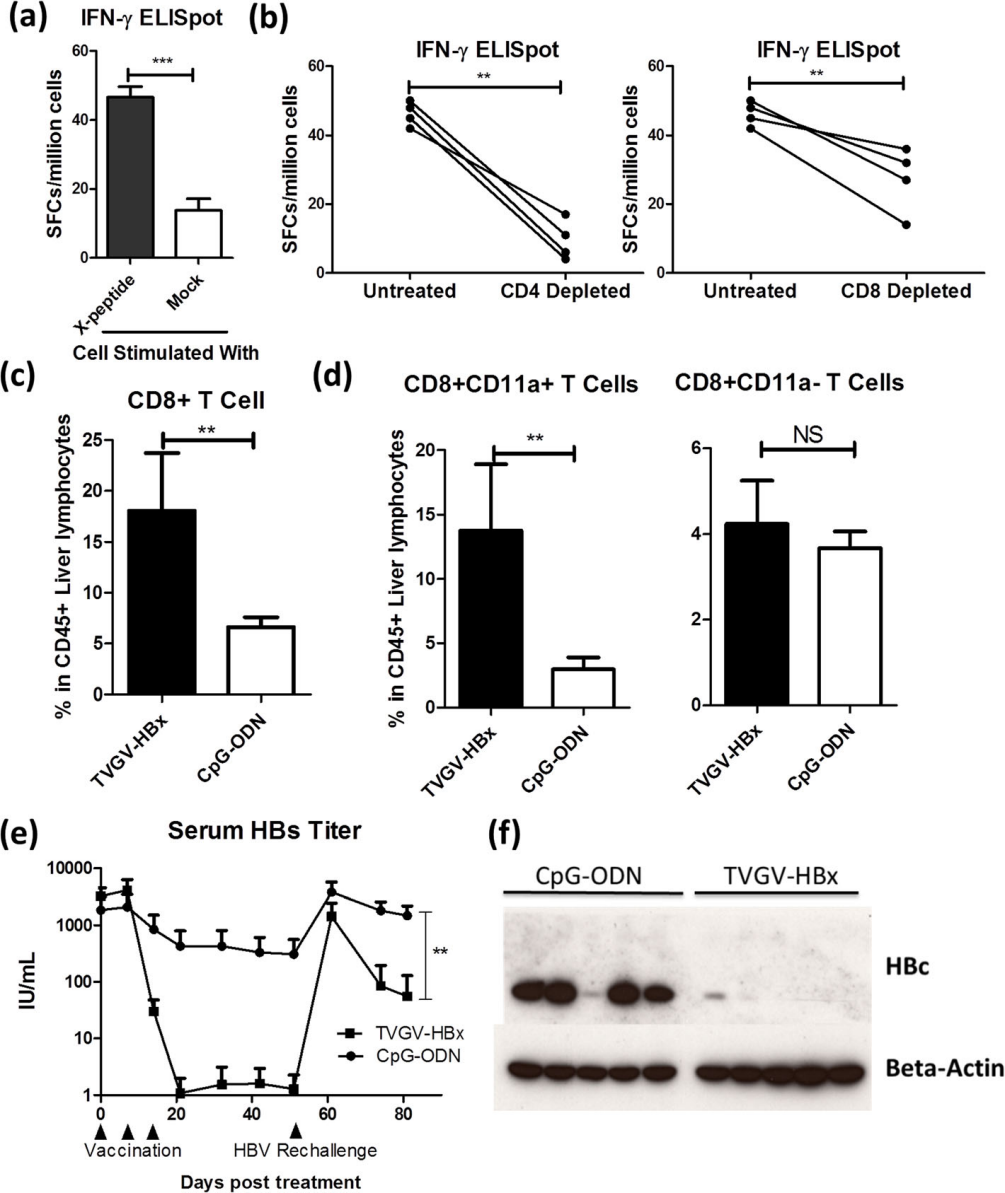
TVGV-HBx induces an HBx-specific T cell response and posttreatment immune memory in HBV carrier mice. **a**-**b** HBV carrier mice (*N* = 4) received TVGV-HBx on days 0, 7 and 14. Splenocytes were isolated at day 21 and subjected to ex vivo CD4^+^ or CD8^+^ T cell depletion or left untreated. Splenocyte preparations were subjected to 15-mer overlapping HBx peptide-stimulated IFN-γ ELISpot assays. **a** Normalized spot counts of untreated splenocytes. **b** Pairwise comparative results for untreated splenocytes versus CD4^+^ cell- or CD8^+^ cell-depleted splenocytes. **c**-**d** HBV carrier mice (N = 3 ~ 4) received TVGV-HBx or CpG-ODN alone on days 0 and 7. Intrahepatic lymphocytes were isolated on day 10. Isolated cells were analyzed by flow cytometry to evaluate T cell subpopulations. **c** Frequency of liver CD8^+^ T cells among total CD45^+^ intrahepatic lymphocytes. **d** Frequency of liver CD11a^+^CD8^+^ and CD11a^−^CD8^+^ T cells among CD45^+^ intrahepatic lymphocytes. **e**-**f** HBV carrier mice (N = 5) were immunized with TVGV-HBx or CpG-ODN on days 0, 7 and 14 and rechallenged with HDI of 10 μg of pAAV/HBV1.2 plasmid on day 53. Blood samples were collected at the time points indicated on the plots, and liver samples were collected on day 84. **e** Serum HBs titer were determined. **f** Liver HBc was detected by western blotting. Statistics: Student’s *t*-test. **, p < 0.01; ***, p < 0.001; NS, not significant; SFCs, spot-forming cells

**Fig. 4:**
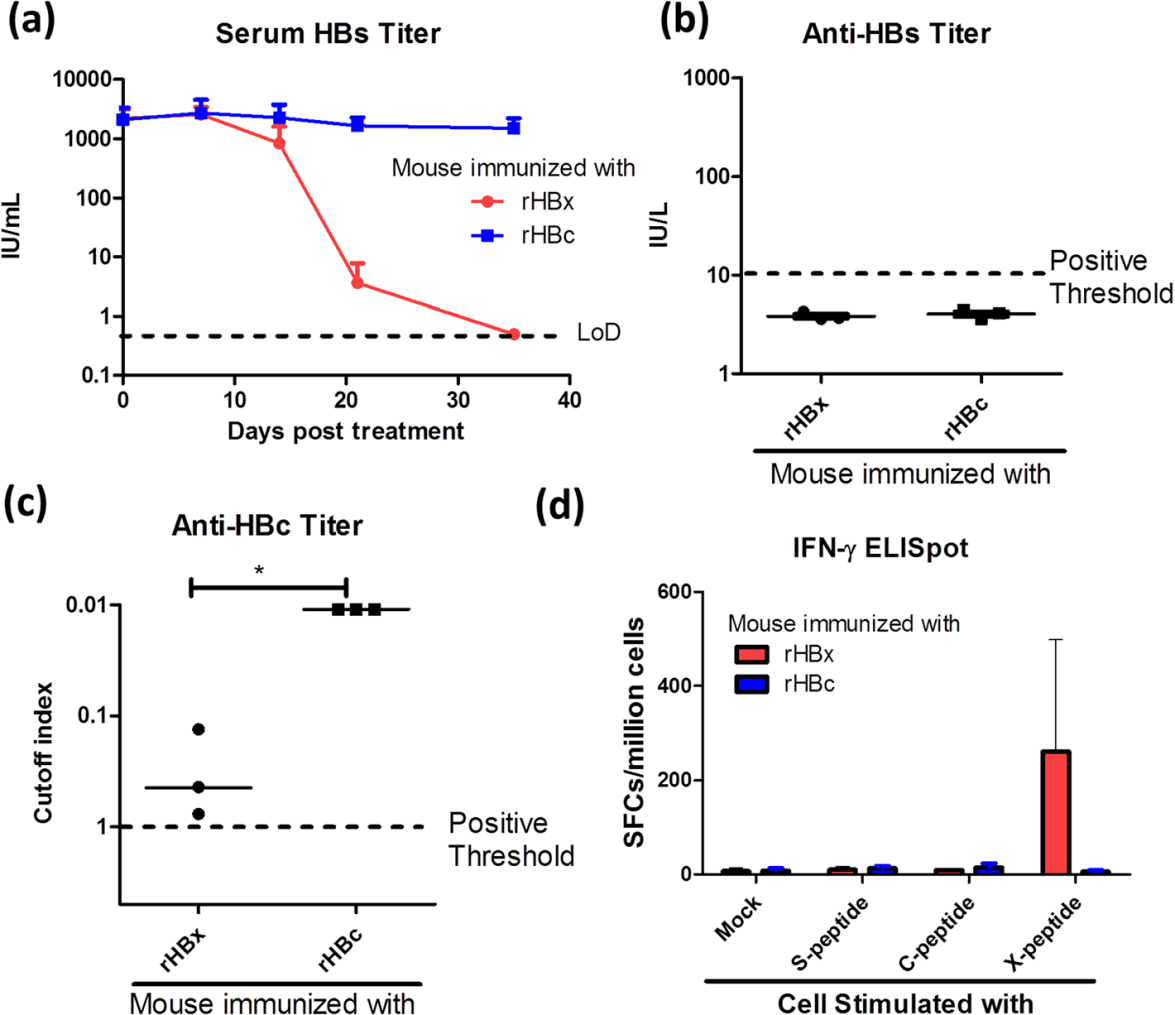
Comparison of the immunogenicity of HBV core antigen and that of HBV X protein. **a**-**c** HBV carrier mice (N = 3) received vaccination with 3.125 μg of rHBc or rHBx supplemented with 0.625 μg of CpG-ODN on days 0, 7 and 14. Mouse serum was sampled on days 0, 7, 14, 21 and 35. (a) Serum HBs titers were determined. **b** Serum anti-HBs antibody titer on day 35. **c** Serum anti-HBc antibody titer on day 35. Lower values represent higher antibody production because the anti-HBc assay is a competition assay. **d** HBV carrier mice (N = 3 ~ 4) received vaccination with 6.25 μg of rHBc or rHBx supplemented with 1.25 μg of CpG-ODN on days 0, 7, 14 and 22. Mice were boosted once with the same dose 7 days before splenocyte isolation. Splenocytes were separately stimulated with HBx-, HBs-, or HBc-derived 15-mer overlapping peptide pool and subjected to an IFN-γ ELISpot assay. The result was normalized to the spot counts per million cells. LoD, limit of detection; SFCs, spot-forming cells

**Fig. 5:**
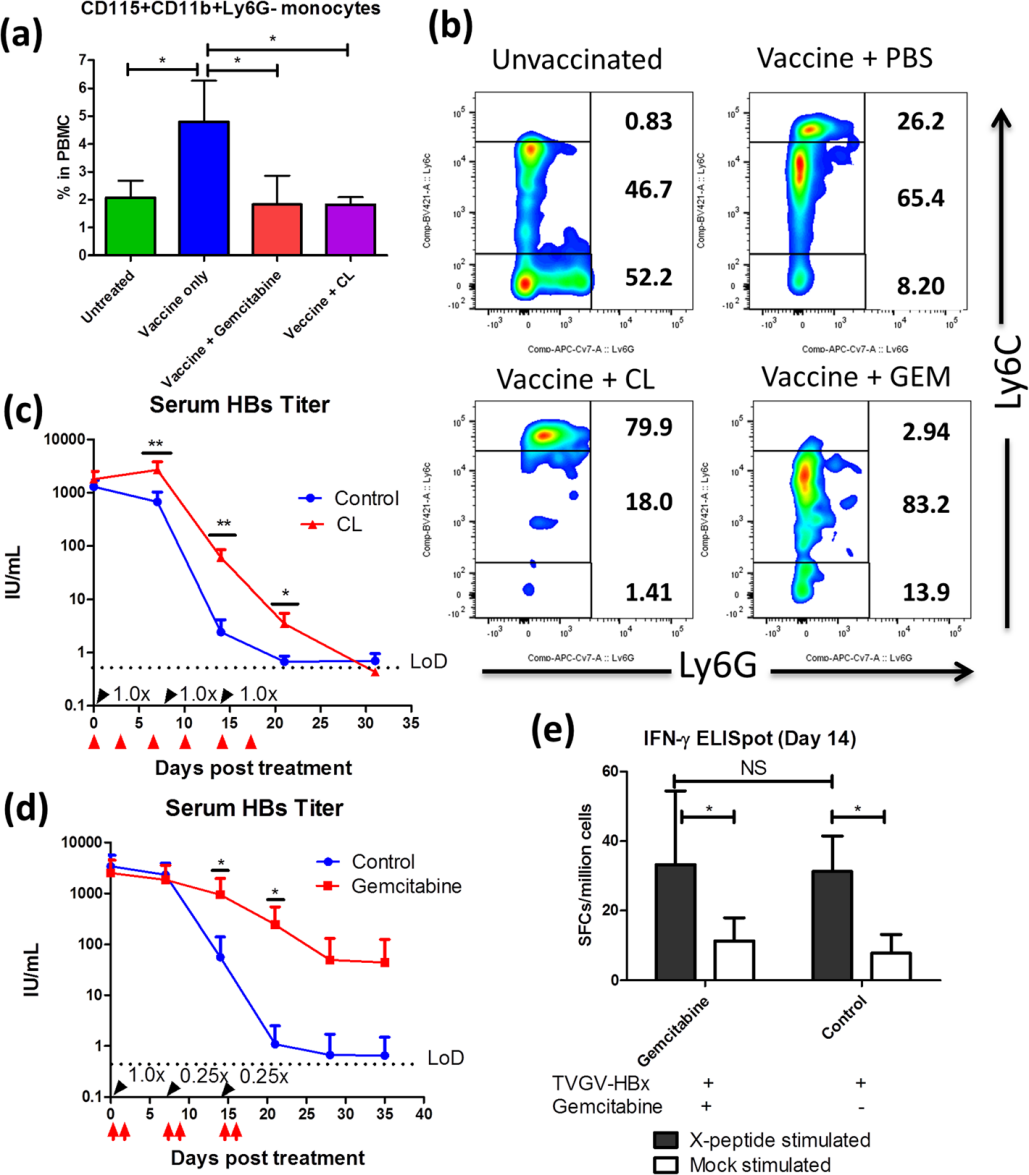
CpG-ODN-induced monocyte mobilization regulates TVGV-HBx-mediated HBV clearance. **a**-**b** HBV carrier mice (N = 3) received TVGV-HBx on day 0. Gemcitabine (40 mg/kg) was given by intraperitoneal injection on days 0 and 1. CLs (200 μL) were given by intravenous injection on day 0. PBMCs were isolated on day 2. PBMCs were stained with fluorochrome-conjugated anti-CD11b, anti-CD115, anti-Ly6C and anti-Ly6G antibodies and then subjected to FACS analysis. **a** Frequency of CD11b^+^CD115^+^Ly6G^−^ monocytes among total PBMCs. **b** Representative FACS plots of Ly6C^+^ monocyte subsets (gated by CD11b + CD115 + Ly6G-) before and after vaccination and drug administration. The cell frequencies of each subpopulation are shown on the plot. **c**-**d** HBV carrier mice (N = 4 ~ 5) were administered TVGV-HBx vaccination (black arrow with the dosage indicated, 100 μg of antigen plus 20 μg of CpG-ODN as 1.0x) and a monocyte-depleting drug (red arrow) at the indicated time points. Blood samples were collected at the indicated time points. **c** Serum HBs titer after TVGV-HBx vaccination and CL treatment. **d** Serum HBs titer after TVGV-HBx vaccination and gemcitabine treatment. **e** Splenocyte IFN-γ ELISpot results (mice N = 5) after TVGV-HBx vaccination and gemcitabine treatment. The vaccination and drug administration protocol were the same as in (**d**), and the splenocytes were harvested on day 14. Cells were stimulated with HBx-derived 15-mer overlapping peptides. Statistics: Student’s *t*-test. *, p < 0.05; **, p < 0.01; NS, not significant; LoD, limit of detection; SFCs, spot-forming cells; GEM, gemcitabine

Mouse whole blood was collected in a plastic microcentrifuge tube and centrifuged at 13,000×g to obtain the serum. The serum was diluted 10-fold in PBS and then analyzed with the following methods: HBs was analyzed with an Elecsys HBsAg II kit (Roche; Fig. 1a) or Architect HBsAg QT assay (Abbott, Lake Forest, IL, USA; Figs. 1c, 2a, 3e, 4a, 5c and d); anti-HBs antibodies were analyzed with an Elecsys Anti-HBs II kit (Roche); and anti-HBc antibodies were analyzed with an Elecsys Anti-HBc II kit (Roche).

### Western blotting

Total liver lysates were prepared by homogenizing 50 mg of liver tissue in 1 mL of T-PER™ reagent supplemented with a phosphatase and protease inhibitor cocktail (Thermo Fisher Scientific). The lysates were cleared by centrifugation at 13,000×g for 15 min. A total of 90 μg of liver protein lysate was separated by 12% sodium dodecyl sulfate polyacrylamide gel electrophoresis and transferred to a PVDF membrane. The membrane was blocked with 5% nonfat milk for 1 h at room temperature. The membrane was probed for HBc with a rabbit anti-HBc polyclonal antibody (clone P3-NP, produced in our laboratory) overnight at 4 °C. An anti-mouse beta-actin antibody (Sigma-Aldrich, St. Louis, MO, USA) served as an internal control. Protein signals were visualized with an appropriate horseradish peroxidase-conjugated secondary antibody (Bio-Rad, Hercules, CA, USA) and enhanced chemiluminescence substrate (Bio-Rad).

### IFN-γ enzyme-linked immunospot assay

Mouse splenocytes were collected by mincing the spleen through 70-μm nylon mesh. Red blood cells were lysed by ACK lysis buffer (Thermo Fisher Scientific). In the ex vivo T cell-depletion experiment, splenocytes were further labeled with a phycoerythrin-conjugated anti-CD4 antibody or an APC-conjugated CD8-specific antibody (BioLegend, San Diego, CA, USA). Fluorochrome-labeled cells were then depleted with anti-phycoerythrin or anti-APC IMAG-magnetic microbeads (BD Bioscience, San Jose, CA, USA) according to the manufacturer’s protocol. The enzyme-linked immunospot (ELISpot) assay was conducted by using the Mouse IFN-γ ELISpot Kit (BD Bioscience) according to the manufacturer’s protocol. A total of 0.3 million splenocytes were seeded in an anti-IFN-γ antibody-coated PVDF plate. The cells were cultured with 10% fetal bovine serum (FBS) supplemented RPMI-1640 medium, and then stimulated with synthetic 15-mer overlapping peptide pools (JPT Peptide Technologies, Berlin, Germany) spanning the whole protein sequence at the concentration of 1 μg/mL per peptide for 18 h. Spots were visualized with an AEC substrate kit (BD Bioscience) according to the manufacturer’s protocol. Spot counting and analysis were conducted with an immunospot analyzer (Cellular Technology, Cleveland, OH, USA).

### Mouse liver lymphoid and myeloid cell isolation

For mouse liver-resident lymphoid cell isolation, the liver was perfused with 5 mL of PBS to remove blood cells. The liver was isolated and then minced by passage through a 100-μm nylon mesh (BD Bioscience) to obtain a liver cell suspension. The cell suspension was centrifuged at 50×g twice to remove hepatocytes, and the crude lymphoid cells in the supernatant were pelleted by 300×g centrifugation. The cell pellet was re-suspended in 4 mL of 40% Percoll solution (GE Healthcare, Chicago, IL, USA), which was then layered on top of 4 mL of 70% Percoll solution. The gradient was separated by 1200×g centrifugation at 25 °C for 20 min with the brake off. The lymphoid cells were recovered from the 40%/70% Percoll interphase and washed with PBS.

The mouse liver myeloid cell isolation protocol was based on the method published by Wu et al. [22] with minor modifications. Mice were deeply anesthetized prior to opening the abdomen to expose the portal vein and inferior vena cava. The liver was perfused with 50 mL of Ca^2+^/Mg^2+^-free Hank’s balanced salt solution (HBSS), followed by perfusion of 40 mL of 0.05% collagenase (Sigma-Aldrich) solution in Ca^2+^/Mg^2 + −^containing HBSS through the portal vein at 37 °C with a 5 mL/min flow rate. The collagenase-digested liver was isolated and dispersed in 50 mL of Ca^2+^/Mg^2 + −^containing HBSS supplemented with 3% FBS to release intrahepatic cells. The cell suspension was centrifuged at 50×g twice to separate hepatocytes and crude hepatic NPCs. The hepatic NPCs in the supernatant were pelleted by centrifugation at 300×g. The crude NPCs were labeled with a phycoerythrin-conjugated anti-mouse CD45 antibody, and the CD45^+^ leukocytes were positively selected with anti-phycoerythrin IMAG-magnetic microbeads (BD Bioscience) according to the manufacturer’s suggestion. Both isolated lymphoid cells and isolated myeloid cells were resuspended in 0.1 mL of PBS containing 3% FBS for further analysis. Total isolated cell counts were calculated with a hemocytometer under a microscope.

### Mouse peripheral blood mononuclear cell isolation and the complete blood count

Mouse whole blood was collected in K2-EDTA tubes (BD Bioscience). Blood samples (100 μl) were subjected to red blood cell lysis by adding 4 mL of ACK lysis buffer (Thermo Fisher Scientific) and incubated for 5 min. After neutralization with 30 mL of PBS, mouse PBMCs were pelleted by centrifugation at 300×g and resuspended in 0.1 mL of PBS containing 3% FBS for further analysis. Complete blood count analysis was conducted with the ProCyte Dx Hematology Analyzer (IDEXX, Westbrook, ME, USA) with 100 μL of whole blood.

### Flow cytometry analysis

Isolated liver leukocytes or PBMCs were blocked with 5 μg/mL anti-mouse CD16/CD32 antibodies (BD Bioscience) and stained with fluorochrome-conjugated antibodies for 30 min at 4 °C in the dark. The cells were washed twice with PBS containing 3% FBS and fixed in 0.5 mL of Fluorofix buffer (BioLegend) for 30 min. The fixed cells were washed with PBS containing 3% FBS once and then analyzed on a FACSFortessa instrument (BD Bioscience). The fluorochrome-conjugated antibodies used in this study included FITC-conjugated anti-CD3, FITC-conjugated anti-CD11b, phycoerythrin-conjugated anti-CD45, APC-conjugated anti-CD8, APC-conjugated anti-CD115, APC/Cy7-conjugated anti-CD45, APC/Cy7-conjugated anti-Ly6G, BV605-conjugated anti-CD4, BV605-conjugated anti-Ly6G, BV421-conjugated anti-Ly6C, PerCP/Cy5.5-conjugated anti-CD19, PerCP/Cy5.5-conjugated anti-F4/80 (all purchased from BioLegend) and BV421-conjugated anti-CD11a (BD Bioscience). The compensation settings of the flow cytometer were determined with single-stained Ultracomp eBeads (Thermo Fisher Scientific). The frequency of each cell subpopulation obtained by flow cytometric analysis was further multiplied by the total isolated cell count to obtain the cell number for each subpopulation.

### Administration of monocyte-depleting drugs

Clodronate liposomes (CLs) are a substance with selective toxicity to phagocytic mononuclear cells, including phagocytic monocytes and macrophages. CLs (Encapsula NanoSciences, Brentwood, TN, USA) or control liposomes were given at 200 μL per mouse via intravenous tail vein injection. Administration was conducted twice per week for 3 weeks. Gemcitabine is a DNA synthesis inhibitor that has been reported to selectively deplete Ly6C-high inflammatory monocytes (Ly6C^high^ monocytes) [23]. Gemcitabine (Sigma-Aldrich) was dissolved in PBS, and a 40 mg/kg dose was given via intraperitoneal injection on days 0 and 1 after every vaccination dose.

### Statistical analyses

All statistical analyses were performed using Prism 5 software (GraphPad, San Diego, CA, USA). All data are presented as the mean and standard deviation. The significance of differences between groups was assessed by Student’s *t*-test. Symbols: * *p* < 0.05; ** *p* < 0.01; *** *p* < 0.001.

## Results

### Potent immunogenicity of an HBx-based therapeutic vaccine in CBA/CaJ mice

To investigate the potency of an HBx-based, CpG-ODN-adjuvanted novel therapeutic vaccine formulation, namely, TVGV-HBx, in an animal model that mimics human HBV carriers, we utilized a previously established hydrodynamic injection (HDI) based model [20] that has been widely adopted for HBV-related vaccine studies [24, 25]. We chose a specific mouse strain, CBA/CaJ, because it showed the highest HBV persistence rate and viral titer after HDI-based pAAV/HBV1.2 plasmid transfection among all tested mouse strains [26]. We first studied the persistence rate of 3 different genotypes (A, B and C) of the pAAV/HBV1.2 plasmid in CBA/CaJ mice. After a single HDI, HBs remained persistent in the mouse blood for more than 6 months without anti-HBs antibody production (Fig. 1a and Supplementary Figure 2). Because the genotype A plasmid resulted in the highest persistence rate, all subsequent experiments were performed using the genotype A plasmid unless otherwise indicated.

We next examined the immunogenicity of TVGV-HBx in naïve mice. Three weekly doses of TVGV-HBx, TVGV-E7 (an HPV-E7 control vaccine) or PBS were separately administered to naïve mice. As it is difficult to reliably assay the antibody responses to HBx, we studied HBx-specific T cell responses instead. Splenocytes from each group were subjected to an IFN-γ ELISpot assay 7 days after the last immunization. The TVGV-HBx-immunized group exhibited a significant increase in IFN-γ secretion in response to HBx-derived peptide stimulation but not in response to HBc-derived peptide stimulation or mock stimulation (Fig. 1b). The TVGV-E7-immunized and unimmunized mice did not exhibit significant HBx-specific T cell responses. These data suggested that TVGV-HBx immunization induced sufficient HBx-specific T cell immunity in naïve mice.

To study whether TVGV-HBx induces protection against HBV, mice received 3 weekly doses of TVGV-HBx and CpG-ODN then followed by HDI pAAV/HBV1.2 plasmid challenge 7 days after the last immunization. We examined the HBV titer by measuring serum HBs and HBV-DNA levels in vaccinated mice at the indicated time points (Fig. 1c and d). The serum HBs level of the TVGV-HBx-immunized mice was approximately 100-fold lower than that of the mice immunized with only the adjuvant CpG-ODN at 28 days post HDI (Fig. 1c). Similar to the HBs level, the serum HBV-DNA titer rapidly decreased to undetectable levels within 2 weeks after pAAV/HBV1.2 plasmid injection in the TVGV-HBx vaccinated group, while the level in the adjuvant group remained high (Fig. 1d). Compared with the CpG-ODN group, the TVGV-HBx-vaccinated group showed a significant reduction in the liver HBc protein level (Fig. 1e). The liver HBV-DNA level was also reduced by approximately 60-fold on average (Fig. 1f). This suggests that prophylactic administration of the TVGV-HBx vaccine induces a protective immune response against HBV that removes both HBV proteins and DNA templates from mouse liver challenged with pAAV/HBV1.2 plasmid.

### TVGV-HBx vaccination clears HBs and HBV-DNA in HBV carrier mice

After confirming the immunogenicity of the TVGV-HBx vaccine in naïve mice, we examined whether the TVGV-HBx vaccine could eradicate HBV (HBV genotype C; Fig. 2a-e) in carrier mice. Different vaccine formulations, including TVGV-HBx, TVGV-E7, CpG-ODN and PBS alone, were each administered to HBV carrier mice weekly for 3 weeks. The therapeutic efficacies of the vaccine formulations were evaluated by determining the clearance rates of serum HBs and HBV-DNA. As a result, compared with the PBS-, CpG-ODN-, and TVGV-E7-vaccinated groups, the TVGV-HBx-treated group displayed rapid and significant reductions in serum HBs and HBV-DNA levels (Fig. 2a and b), indicating that therapeutic TVGV-HBx vaccination was able to dramatically decrease serum viral titers in HBV carrier mice. However, after HBs was cleared from the serum, we did not find protective anti-HBs antibody production in the serum (Fig. 2c). This result suggested that immunization with the HBx vaccine alone did not restore anti-HBs antibody production and that the removal of HBs from the serum was not due to a neutralizing antibody. At the endpoint of the experiment, all TVGV-HBx-vaccinated HBV carrier mice exhibited undetectable liver HBc and HBV-DNA levels, demonstrating complete HBV clearance from the liver (Fig. 2d and e). These findings suggested that TVGV-HBx immunization was able to eliminate preexisting HBV-expressing hepatocytes in HBV carrier mice. Furthermore, compared with the PBS group, both the CpG-ODN-treated groups (TVGV-E7 with the adjuvant CpG-ODN and CpG-ODN alone) showed a 2- to 4-fold decrease in the serum HBs level (Fig. 2a), and a couple of mice (2/12, 17%) achieved liver HBV-DNA clearance (Fig. 2e). These results indicate that the adjuvant CpG-ODN may trigger innate immune responses that partially contribute to HBV clearance. Similar TVGV-HBx therapeutic efficacy were obtained with HBV carrier mice injected with other HBV genotypes (A, B, and D), indicating that the TVGV-HBx-induced antiviral activity was not limited to a specific HBV genotype (Supplementary Figure 3a-c).

### TVGV-HBx vaccination induces an HBx-specific T cell response and posttherapy protective effects in the context of HBV viremia

High antigen loads have been reported to inhibit effector T cell function, which is a key factor in viral clearance. Splenocytes were collected from HBV carrier mice after immunization with 3 weekly doses of TVGV-HBx to assess the efficacy of TVGV-HBx vaccination in inducing systemic T cell-mediated immunity in the context of HBV viremia. HBx-specific T cell ELISpot assay results revealed that TVGV-HBx-immunized carriers exhibited a specific response to an HBx-derived peptide, but those in the mock-stimulated group did not (Fig. 3a). To determine whether the TVGV-HBx-induced T cell response involves CD4^+^ or CD8^+^ subpopulations, in the same experiment, we predepleted CD4^+^ or CD8^+^ T cells during splenocyte preparation and compared the resultant ELISpot results with those for nondepleted splenocytes. The results showed that both CD4^+^ cell- and CD8^+^ cell-depleted splenocytes demonstrated a significant decrease in the TVGV-HBx-induced T cell response, with the drop in the CD4^+^ cell-depleted group being greater than that in the CD8^+^ cell-depleted group (Fig. 3b). To confirm whether vaccine-induced cytotoxic T cells infiltrate the liver, we analyzed the hepatic CD8^+^ T cell quantity at day 10 when the serum HBs titer was descending (Fig. 2a and Supplementary Figure 3a-c). We found that compared to that in the CpG-ODN alone group, the CD8^+^ T cell number in the liver in the TVGV-HBx-immunized group was significantly increased (Fig. 3c). In addition, we also found that most of the liver CD8^+^ T cells in the increased population were CD11a^+^CD8^+^ cells (Fig. 3d), which have been reported to be antigen-experienced cytotoxic T cells that are important for hepatic HBV clearance [24, 27]. These findings signify that TVGV-HBx immunization is able to induce systemic HBx-specific CD4^+^ and CD8^+^ T lymphocytes in HBV carrier mice and that vaccine-induced CD8^+^ T lymphocytes will infiltrate the liver to eliminate HBV.

After confirming that the TVGV-HBx vaccine could clear HBV in the liver, we further investigated whether the TVGV-HBx vaccine is able to provide a long-lasting protective effect against a second HBV exposure after the initial cure. We immunized HBV carrier mice with 3 weekly doses of TVGV-HBx or the adjuvant alone. At day 32 after the first HBV clearance, we re-challenged all animals with the same pAAV/HBV1.2 plasmid by HDI and measured the serum HBs titer weekly. The results showed that without an additional booster immunization, the serum HBs level following the second HBV exposure was significantly reduced by approximately 25-fold in the TVGV-HBx-immunized mice compared with the adjuvant-alone group (Fig. 3e). Western blot analysis of liver tissue confirmed that TVGV-HBx immunization led to the clearance of most HBc after the second HBV exposure (Fig. 3f). These results implied that TVGV-HBx vaccination provided a protective effect after vaccination to accelerate the clearance of HBV after the second exposure. However, the mice could not immediately eliminate serum HBs after the second challenge, suggesting weaker intrahepatic immunity during the second HBV exposure than during the first.

### HBc and HBx, as immunogens, induce distinct types of immunity against HBV

To explore the impact of immunogen selection on the efficacy of therapeutic vaccination in HBV carrier mice, we compared the HBV clearance potency of HBc, a relatively abundant and highly immunogenic structural protein of HBV, with that of HBx, with both proteins used as immunogens. CpG-ODN-adjuvanted recombinant HBx or HBc were administered to HBV carrier mice at a previously titrated minimal effective dosage of TVGV-HBx (Supplementary Figure 4). As expected, the HBx-immunized mice had completely eliminated serum HBs at day 31, whereas HBc immunization exhibited a minimal impact on serum HBs clearance, indicating that HBc is a less effective immunogen than HBx in mice (Fig. 4a). In addition, we also evaluated the status of humoral immunity after immunization with the different antigens by evaluating anti-HBs and anti-HBc antibody production at the endpoint of the experiment. As shown, neither HBc- nor HBx-immunized mice exhibited anti-HBs antibody production (Fig. 4b). All mice were positive for anti-HBc antibodies in the serum, indicating that the hepatocytes expressing HBc in this model could induce baseline antibody production. Among the HBc-immunized mice, the anti-HBc antibody level was higher than that in the HBx-immunized mice and exceeded the detection limit even with a 10-fold dilution (Fig. 4c). This result suggested that HBc immunization potently bolstered anti-HBc antibody production in mice but did not result in HBV clearance. We also evaluated the splenic T cell response against corresponding peptides derived from each antigen, which showed that only HBx immunization, not HBc immunization, resulted in strong induction of antigen-specific IFN-γ-secreting T cells (Fig. 4d). These results imply that the HBx-based vaccine induces better cellular immunity than the HBc-based vaccine. Since antigen-specific T cell response induction is critical to viral clearance, this result was in concordance with the serum HBs clearance rate results indicating that HBx had better immunogenicity, resulting in elimination of persistent HBV, than HBc (Fig. 4a).

### TVGV-HBx vaccination promotes HBV clearance by mobilizing monocytes

To explore possible innate immune modulators that contribute to vaccine-mediated HBV clearance, we monitored the blood leukocyte count changes in TVGV-HBx-, TVGV-E7-, CpG-ODN- and PBS-immunized mice twice a week. Among the counts of all blood leukocyte types, only the monocyte count showed TVGV-HBx-specific changes. All CpG-ODN-containing groups demonstrated elevated monocyte counts at day 3 postvaccination. Interestingly, only the TVGV-HBx-immunized mice showed delayed deflation at day 7 postimmunization (Supplementary Figure 5). Based on this observation, we speculated that vaccine-mobilized monocytes are associated with TVGV-HBx-induced HBV clearance.

Next, we investigated whether removing monocytes impacts the efficacy of TVGV-HBx vaccination. We tested 2 monocyte-depleting drugs, CLs and gemcitabine, in mice and checked their blood monocyte frequency after TVGV-HBx stimulation and drug treatment. We observed that TVGV-HBx immunization strongly mobilized CD11b^+^CD115^+^Ly6G^−^ monocytes into the blood, with especially Ly6C^high^ monocytes showing a dramatic expansion (Fig. 5a and b). After drug administration, compared with the vaccine-only group, the CL and gemcitabine groups each showed an approximately 50% reduction in the CD11b^+^CD115^+^Ly6G^−^ blood monocyte frequency (Fig. 5a). Interestingly, CLs and gemcitabine depleted different monocyte subpopulations according to surface Ly6C expression. CLs mainly depleted Ly6C-intermediate and Ly6C-low monocyte-derived macrophages (Ly6C^int^ and Ly6C^low^ MoMs, respectively), probably due to their selective cytotoxicity to phagocytic cells (Fig. 5b). In contrast, gemcitabine depleted almost all Ly6C^high^ monocytes (Fig. 5b). Neither CLs nor gemcitabine depleted CD11b^+^Ly6G^+^ neutrophils (Supplementary Figure 6). After checking the depletion efficiencies of both drugs, we administered TVGV-HBx immunization to mice pre-treated with CLs or gemcitabine and tested serum HBs clearance. In the CL depletion experiment, we treated animals with CLs twice a week until day 17 to maintain the depletion of phagocytic macrophages during the therapy course and compared this group against the control liposome-treated group. The CL depletion group showed an increased serum HBs level from day 7 to 21 but eventually displayed a modest difference in the HBs clearance profile (Fig. 5c). This result suggests that without phagocytic Ly6C^int^ and Ly6C^low^ MoMs, the presence of Ly6C^high^ monocytes alone is sufficient to support vaccine-induced HBV clearance. To further investigate the significance of Ly6C^high^ monocytes in vaccine-mediated HBV clearance, we treated animals with gemcitabine at days 0 and 1 after every vaccination dose to inhibit vaccine-induced Ly6C^high^ monocyte mobilization and minimize the suppressive effect on proliferating T cells. Mice treated with gemcitabine showed significantly delayed HBs clearance from day 14 to 21, which resulted in a 60-fold higher serum HBs level in the treated group than in the control group at the endpoint of the experiment (Fig. 5d). None of the gemcitabine-treated mice achieved complete HBV clearance. Since gemcitabine is a DNA synthesis inhibitor that could possibly inhibit T cell proliferation in the secondary lymphoid organs, we conducted splenic IFN-γ ELISpot experiments on days 14 and 21 with the same administration protocol to determine whether systemic T cell immunity is affected by gemcitabine treatment. As indicated by the results, gemcitabine-treated mice did not exhibit deficiencies in systemic HBx-specific T cell immunity, suggesting that the inhibitory effect of gemcitabine treatment cannot be attributed to systemic T cell suppression (Fig. 5e and Supplementary Figure 7).

### CL and gemcitabine treatments alter the liver immune environment after vaccination and impacts HBs clearance

**Fig. 6:**
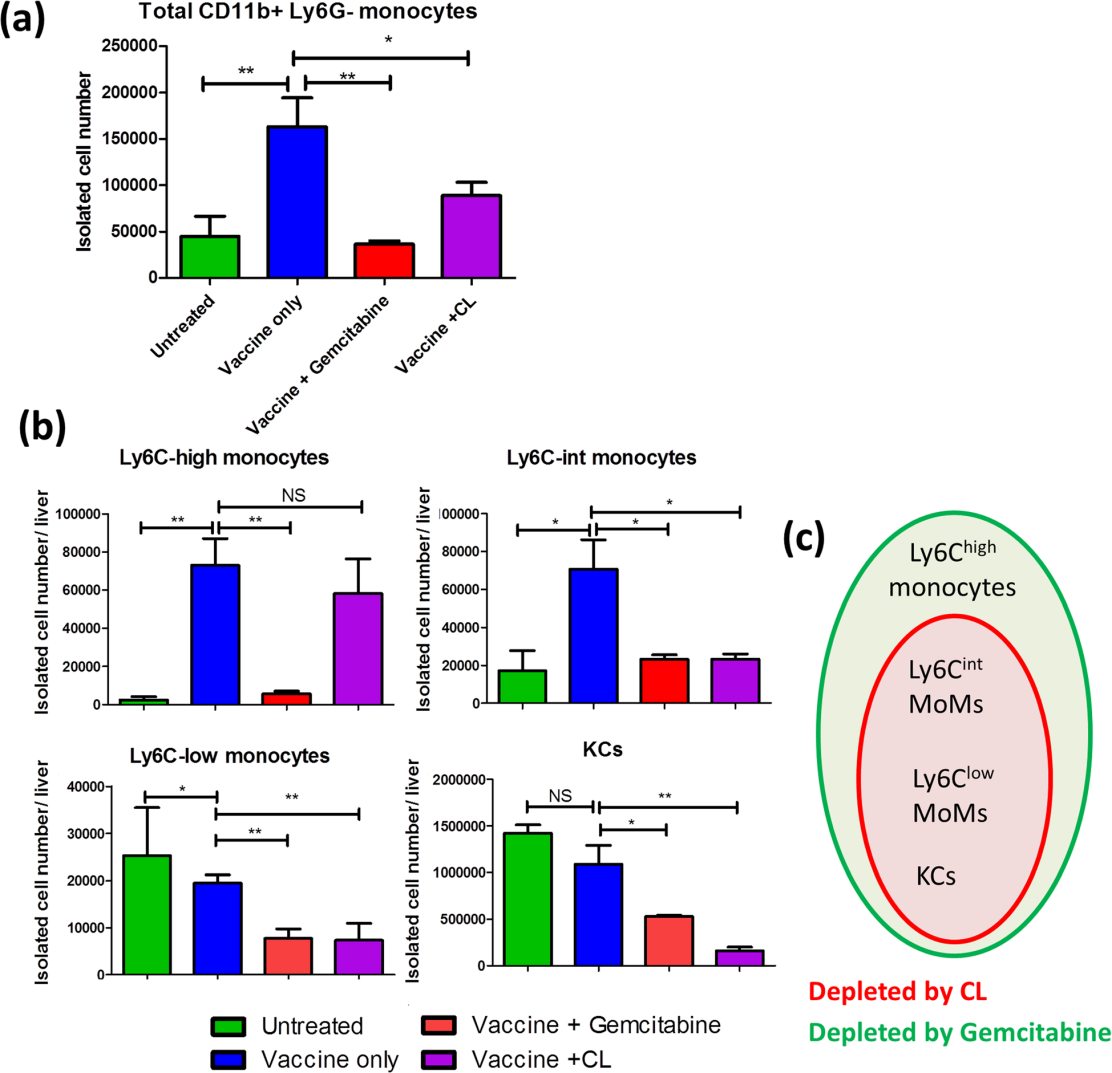
Liver monocyte subpopulation changes after TVGV-HBx vaccination and monocyte-depleting drug treatment. **a**-**b** HBV carrier mice (*N* = 4) received TVGV-HBx on day 0. Gemcitabine (40 mg/kg) was given by intraperitoneal injection on days 0 and 1. CLs (200 μL) were given by intravenous injection on day 0. Total intrahepatic leukocytes were collected on day 2 and subjected to flow cytometric analysis. The isolated cell count of each cell subpopulation was calculated by multiplying the actual isolated CD45^+^ leukocyte count per liver by the subpopulation frequency among isolated cells. **a** Total isolated cell count of all F4/80^low^CD11b^+^Ly6G^−^ monocytes. **b** Total isolated cell counts of the indicated monocyte subsets and F4/80^high^CD11b^+^ KCs. **c** Schematic Venn diagram of liver myeloid cell subpopulations depleted by CL or gemcitabine treatment. Statistics: Student’s *t*-test. *, p < 0.05; **, p < 0.01; NS, not significant

Based on our previous observation that gemcitabine suppresses HBV clearance without affecting systemic immunity, we speculated that gemcitabine exerts an inhibitory effect by interfering with liver-resident innate immunity. We then investigated whether CpG-ODN-mobilized monocytes infiltrate the liver and whether their infiltration is affected by CL or gemcitabine treatment. On day 2 postvaccination, we isolated total intrahepatic leukocytes from TVGV-HBx-immunized carrier mice and then compared their monocyte frequency and absolute cell counts to that of unimmunized mice (Supplementary Figure 8). First, we noted a prominent liver monocyte infiltration after vaccination (Fig. 6a). Most of these cells were found to be Ly6C^high^ monocytes and Ly6C^int^ MoMs, whereas the numbers of Ly6C^low^ cells and Kupffer cells (KCs) did not increase (Fig. 6b). After drug treatment to TVGV-HBx vaccinated mice, both the CL and gemcitabine groups showed a reduced total monocyte number in the liver (Fig. 6a). CLs mainly depleted phagocytic macrophages, including Ly6C^int^ and Ly6C^low^ MoMs and KCs, and had no impact on Ly6C^high^ monocytes (Fig. 6b). On the other hand, gemcitabine had the strongest influence on liver Ly6C^high^ monocytes, and the reductions in KC and Ly6C^int^ and Ly6C^low^ MoM counts were similar to those seen with CL treatment (Fig. 6b). Overall, CLs, in comparison to gemcitabine, did not deplete Ly6C^high^ monocytes (Fig. 6c). The long-term inhibitory effect on HBV clearance occurred only when hepatic Ly6C^high^ monocytes were depleted (Fig. 5c and d). Collectively, our results implied that Ly6C^int^ and Ly6C^low^ MoMs or KCs might be transiently involved in the early stage but were not the deciding factor in vaccine-mediated HBV clearance. In contrast, liver-infiltrating Ly6C^high^ monocytes might play more important roles in TVGV-HBx-induced immune clearance.

## Discussion

Our study shows that our CpG-ODN-adjuvanted HBx-based vaccine is able to induce an effective HBx-specific T cell response, including both CD4^+^ and CD8^+^ subpopulations, to remove persistent HBV genome templates from the HBV carrier mouse liver. As described previously, we chose the low-expressed viral HBx as the antigen because it may have advantages in avoiding excessive antigen-induced T cell exhaustion. However, using a low-expressed intracellular antigen has other potential disadvantages, such as the low-expressed antigen potentially reducing the level of the class I MHC-peptide complex on hepatocytes and decreasing the ability of CD8^+^ T cells to recognize target cells. The low HBx transcript expression levels found in our animal models in this study are similar to those in HBV-infected primary human hepatocytes [14, 28]. Under such conditions, TVGV-HBx-induced adaptive immunity was still able to eliminate HBV-expressing hepatocytes, supporting the feasibility of using HBx as a therapeutic target in CHB. On the other hand, the protein expression of HBx is highly associated with HBc in HBV-infected hepatocytes [29]. Since HBc is necessary for producing new infectious viral particles, elimination of HBx-expressing hepatocytes also removes the source of infectious viral particles, which makes HBx a reasonable therapeutic target. Moreover, after TVGV-HBx treatment, we found that residual protective immunity could last for at least 30 days to reduce the HBV titer after a second HBV exposure, suggesting the existence of immune memory or long-lived effector T cells. However, this immune response was weaker than that observed directly after therapeutic immunization, probably owing to the lack of CpG-ODN-mediated local immune modulation during hepatic immune clearance. As reported in another study, prime-and-boost vaccination alone is not sufficient to clear HBV unless the vaccine is boosted with CpG-ODN [30].

We found that antigen selection strongly affected the type of adaptive immunity induced. When supplemented with CpG-ODN, HBx immunization induced a large amount of antigen-specific T cells and cleared HBV in carrier mice. In contrast, CpG-ODN-adjuvanted HBc did not show significant induction of antigen-specific T cells but instead induced a high level of anti-HBc antibody production. This result is in concordance with a previous vaccine study conducted in HBV transgenic mice [31]. This phenomenon might result from the preferential uptake and presentation of naked HBc by B cells instead of dendritic cells or macrophages [32]. Another possibility is that mouse HBc-specific T cells are already exhausted prior to immunization.

We were unable to directly detect anti-HBx antibodies due to the lack of a reliable assay, but recognition of an intracellular protein by antibodies is not expected to have important roles in HBV immune clearance. No signs of protective anti-HBs antibody recovery were observed after the clearance of serum HBs. This result indicates that treatment with the HBx vaccine alone is not sufficient for the restoration of humoral immunity against HBs, despite the recovery of the HBx-specific T cell response. The restoration of anti-HBs antibody production may require additional HBs immunization. The removal of the HBs burden from the circulation may contribute to the recovery of anti-HBs antibody production, but it still requires further investigation.

Although the first priming of antigen-specific T cells in mice treated with peripheral vaccination usually occurs in the secondary lymphoid tissues, including the spleen or local draining lymph nodes, the tolerogenic liver environment hampers T cell-mediated immune clearance in both antigen-specific and nonspecific manners [11, 16, 17]. Hepatic myeloid cells, including GR1^+^ neutrophils, monocytes, and KCs, have been suggested to promote hepatic HBV antigen clearance [18, 33, 34]. However, myeloid cells are highly heterogeneous [35], and the lack of depletion reagents specific for individual subpopulations increases the difficulty of performing functional analyses. We show that TVGV-HBx vaccination induces robust systemic mobilization and liver infiltration of monocytes, including Ly6C^high^ monocytes and Ly6C^int^ MoMs, both of which were previously reported to be participants in intrahepatic myeloid cell aggregates for T cell population expansion [18]. Through CL- and gemcitabine-mediated cell depletion, we discovered distinctive roles for intrahepatic monocyte subpopulations in resolving hepatic HBV exposure. Phagocytic macrophages, including Ly6C^int^MHCII^+^ MoMs, are the major antigen-presenting cells that support intrahepatic T cell proliferation [18]. However, our model suggests that the removal of these cells by CLs is not sufficient to suppress vaccine-induced HBV clearance. In contrast, a sustained suppressive effect on HBV clearance occurred only when Ly6C^high^ monocytes were depleted. Ly6C^high^ monocytes may also support T cell proliferation in the liver. In addition, they are good producers of multiple immunomodulators, such as IL-12, TNF-α, MCP-1, CXCL10, and CCL5, all of which may modulate the tolerogenic hepatic microenvironment toward immune clearance [36, 37]. Among these cytokines, IL-12 also augments NK cell secretion of IFN-γ and promotes NK cell cytotoxicity [38], which may explain why a small number of CpG-ODN-receiving carrier mice also showed HBV load reduction or clearance. Among liver-infiltrating myeloid cells, Ly6C^high^ monocytes might have pivotal roles in preconditioning the liver immune environment toward immune clearance and supporting subsequent infiltrating antigen-specific T cells in exerting their cytotoxic function to eliminate HBV (Supplementary Figure 9). Our results highlight the significance of Ly6C^high^ monocytes in regulating hepatic immunity during CpG-ODN-adjuvanted vaccination.

As has been shown in many other preclinical attempts with therapeutic HBV vaccines, including those containing HBs and HBc, vaccine-receiving animals usually show excellent efficacy in reducing HBs and HBV-DNA titers, even in models that are reportedly “immune tolerant” toward HBV [18, 24, 30, 39]. Unfortunately, to our knowledge, no therapeutic vaccine has convincingly demonstrated clinical benefits in CHB patients. All current HBV immunology-related animal models, including HDI-based and adeno- or adeno-associated virus-transduced models, have limitations in imitating the host immune responses observed in CHB patients. CHB patients usually carry HBV and develop T cell exhaustion over multiple years or even decades, but in animal models, T cell exhaustion develops within approximately 4–6 weeks. Although these animal models show features of T cell exhaustion, including the expression of inhibitory receptors and reduced cytokine secretion in certain HBV-specific T cell clones [40, 41], it is still questionable whether their T cell exhaustion degree is comparable to that of CHB patients. Additionally, since rodents are not natural hosts of HBV, the establishment of a CHB model in immunocompetent mice usually uses alternative transduction methods to substitute for natural infection; therefore, the transduced HBV genome does not evolve or generate variants that escape host immune responses. HBV clearance may be easier when viral proteins have less variation in a mouse model. Although our study provided evidence that the HBx-based vaccine can eliminate HBV-bearing hepatocytes in a mouse model, translation into clinical use still requires further investigation.

## Conclusions

Our study demonstrates that the HBx-based vaccine successfully eliminates persistent HBV in an animal model and has the potential to be developed into a therapeutic vaccine against CHB. The adjuvant CpG-ODN improves HBx-specific T cell functions by mobilizing Ly6C^high^ monocytes to the liver and shifting the hepatic microenvironment toward one favoring immune clearance. The induction of optimal liver Ly6C^high^ monocyte accumulation and the consequent modulatory effect on the hepatic immune environment are key to subverting the tolerogenic liver environment and facilitating T cell-mediated viral clearance. The appropriate immunogen design in combination with optimal hepatic innate immunity modulation might be important considerations for developing next-generation therapeutic vaccines to treat CHB.

## Supplementary information

**Additional file 1: Figure S1.** Structures of RAP1-HBx, RAP1-E7 and pEt32a-HBx; **Figure S2.** HBV carrier mice do not produce protective anti-HBs antibodies; **Figure S3.** HBx vaccine immunization removes serum HBs in mice carrying different HBV genotypes; **Figure S4.** TVGV-HBx exhibits antiviral activity in a dose-dependent manner; **Figure S5.** Quantification of CpG-ODN-induced monocyte changes in the blood; **Figure S6.** The effect of gemcitabine or clodronate liposome treatment on the blood neutrophil frequency; **Figure S7.** Gemcitabine treatment does not inhibit systemic T cell immunity; **Figure S8.** Representative flow cytometry plots of liver myeloid cell subpopulations after TVGV-HBx vaccination and monocyte depletion; and **Figure S9.** Schematic diagram of monocyte preconditioning of the liver immune environment and its impact on HBx therapeutic vaccine efficacy.

## Data Availability

All data generated or analyzed during this study are included in this published article and the supplementary information files.

## Abbreviations

HBV: Hepatitis B virus
CHB: Chronic hepatitis B
HBx: HBV X protein
HBs: HBV surface antigen
IFN: Interferon
HBc: HBV core antigen
PBMC: Peripheral blood mononuclear cell
NPC: Nonparenchymal cell
HDI: Hydrodynamic injection
PBS: Phosphate-buffered saline
HPV-E7: Human papillomavirus E7 protein
PE-A: Pseudomonas Exotoxin A
CpG-ODN: CpG oligodeoxynucleotides
rHBc: Recombinant HBV core antigen
rHBx: Recombinant HBV X protein
Q-PCR: Quantitative polymerase chain reaction
ELISpot: Enzyme-linked immunospot
HBSS: Hank’s balanced salt solution
FBS: Fetal bovine serum
CL: Clodronate liposome
Ly6C^high^ monocyte: Ly6C-high inflammatory monocyte
MoM: Monocyte-derived macrophage
KC: Kupffer cell
