# Supplementary material for: HBV X protein-based therapeutic vaccine accelerates viral antigen clearance by mobilizing monocyte infiltration into the liver in HBV carrier mice
Source: J Biomed Sci. 2020 May 28;27:70. doi: 10.1186/s12929-020-00662-x (PMC7257178; doi:10.1186/s12929-020-00662-x)
Supplement: Supplementary file 1 — Additional file 1: Figure S1. Structures of RAP1-HBx, RAP1-E7 and pEt32a-HBx; Figure S2. HBV carrier mice do not produce protective anti-HBs antibodies; Figure S3. HBx vaccine immunization removes serum HBs in mice carrying different HBV genotypes; Figure S4. TVGV-HBx exhibits antiviral activity in a dose-dependent manner; Figure S5. Quantification of CpG-ODN-induced monocyte changes in the blood; Figure S6. The effect of gemcitabine or clodronate liposome treatment on the blood neutrophil frequency; Figure S7. Gemcitabine treatment does not inhibit systemic T cell immunity; Figure S8. Representative flow cytometry plots of liver myeloid cell subpopulations after TVGV-HBx vaccination and monocyte depletion; and Figure S9. Schematic diagram of monocyte preconditioning of the liver immune environment and its impact on HBx therapeutic vaccine efficacy. [file 12929_2020_662_MOESM1_ESM.pdf]

Supplementary Figures

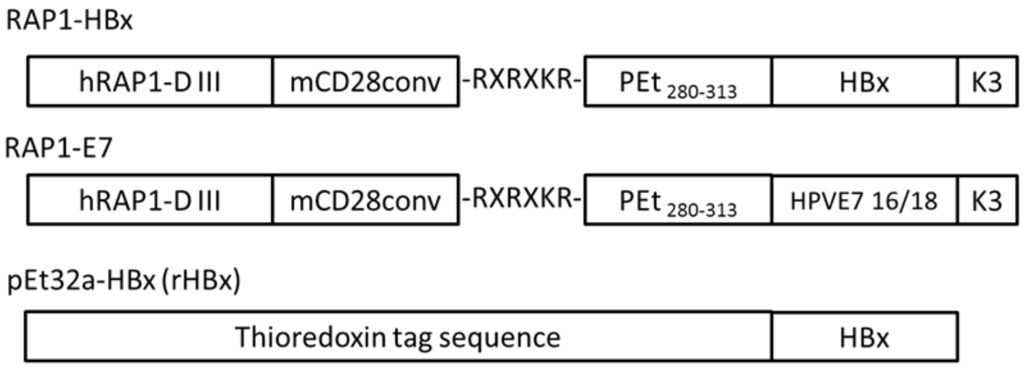

Supplementary figure 1. Structures of RAP1-HBx, RAP1-E7 and pEt32a-HBx

The flanked sequence of HBx and HPV-E7 in the TVGV vaccines are Pheudomonas Exotoxin A (PE-A) mimicry sequence to facilitate antigen processing by professional antigen presenting cells. The thioredoxin tag sequence on rHBx is a non-immune stimulatory control of PE-A sequence. hRAP1-D III, Human RAP1 protein Domain III; mCD28-RXRXKR, linker sequence; pEt$_{280\text{-}313}$-Antigen-K3, sequences facilitate intracellular antigen processing

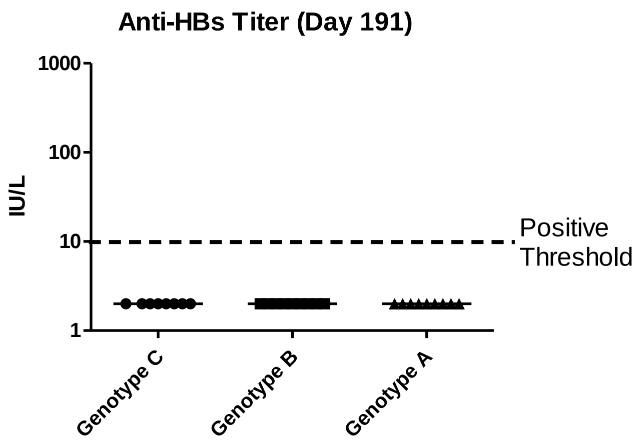

Supplementary figure 2. HBV carrier mice do not produce protective anti-HBs antibodies

Naïve CBA/CaJ mice (N=9) received hydrodynamic injection of 10μg of different genotype pAAV/HBV1.2 plasmid at day 0. Blood samples were collected at day 191, and the serum anti-HBs titers are shown.

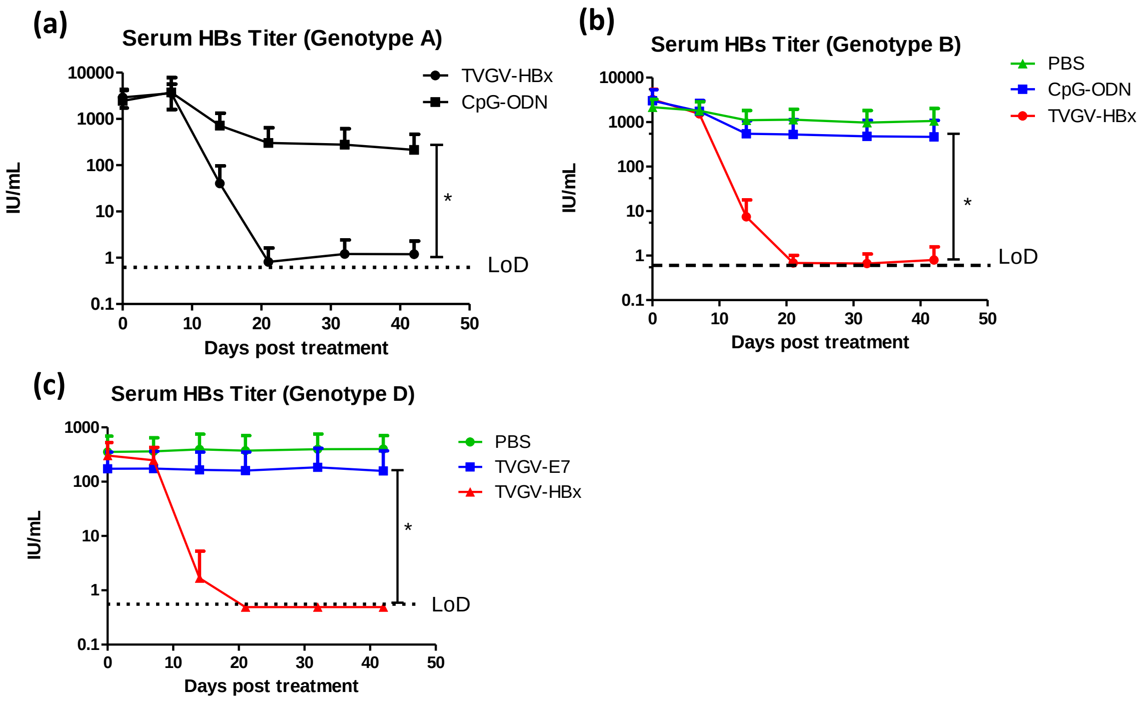

Supplementary figure 3. HBx vaccine immunization removes serum HBs in mice carrying different HBV genotypes

(A)-(C) CBA/CaJ mice (N=9) carrying different HBV genotype including: (A) Genotype A, (B) Genotype B and (C) Genotype D received indicated vaccine or control formula at day 0, 7 and 14. Blood samples was collected at the indicated time points and subjected to serum HBs quantification and the results are shown. Statistics: Student's *t*-test between TVGV-HBx and indicated control groups. LoD, Limit of detection. Symbols: * $p<0.05$.

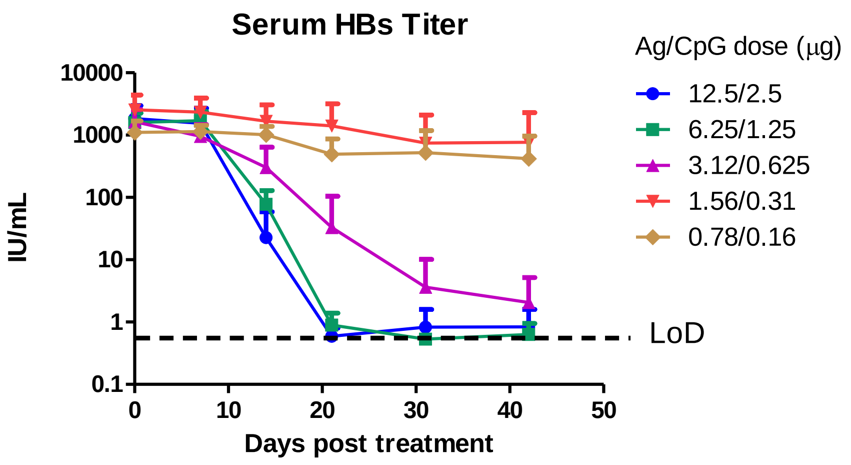

Supplementary figure 4. TVGV-HBx exhibits antiviral activity in a dose-dependent manner

TVGV-HBx (100μg antigen supplemented with 20 μg CpG-ODN) was subject to 2-fold serial dilution to required concentration as indicated. HBV carrier mice (N=4~5) received immunization at day0, 7 and 14 with different doses of TVGV-HBx. The blood sample was collected at the indicated time points, and the serum HBs titers are shown. LoD: Limit of detection.

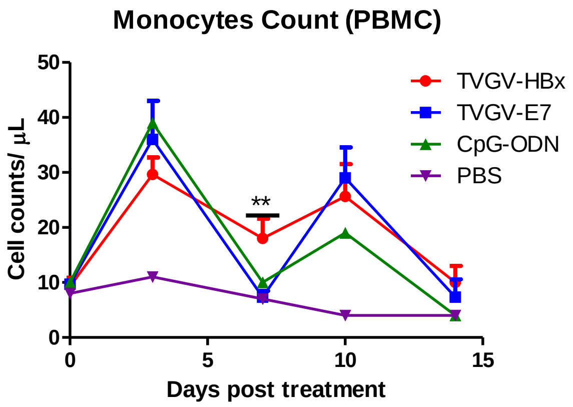

Supplementary figure 5. Quantification of CpG-ODN-induced monocyte changes in the blood

HBV carrier mice (N=3) were immunized with indicated vaccine formula at day 0 and 7. Blood cells were collected at day 0, 3, 7, 10 and 14 and subject to complete blood count assay. The numbers of total blood monocytes are shown in the plot. Statistics: Student's *t*-test between TVGV-HBx and TVGV-E7 group. Symbols: ** $p<0.01$

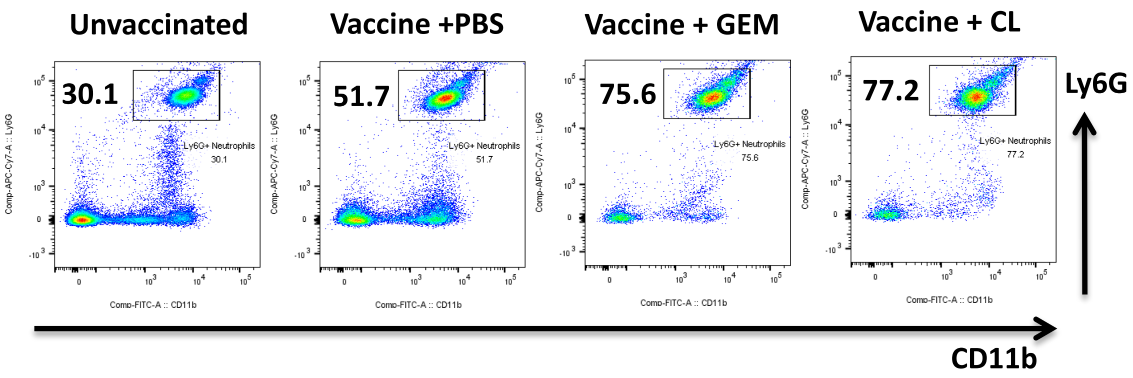

Supplementary figure 6. The effect of gemcitabine or Clodronate liposome treatment on the blood neutrophil frequency

HBV carrier mice (N=3) received TVGV-HBx at day 0. Clodronate liposomes (CLs, 200μL) were given by intravenous injection at day 0. Gemcitabine (GEM, 40mg/kg) was given by intraperitoneal injection at day 0 and 1. The blood leukocytes were isolated at day 2. Neutrophils were gated by $CD11b^+Ly6G^+$ cells and their frequency in PBMC are shown.

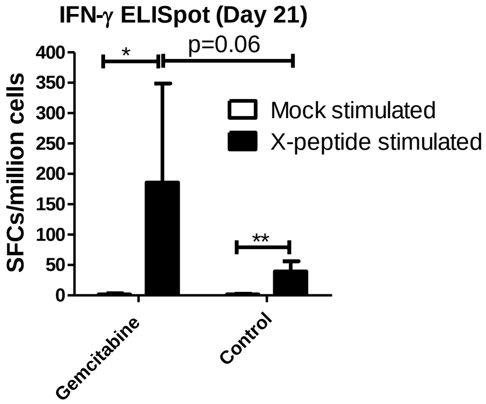

Supplementary figure 7. Gemcitabine treatment does not inhibit systemic T cell immunity

Naïve C57BL/6J mice (N=4) were administrated with standard dose TVGV-HBx at day 0, 7, and 14. Gemcitabine (40mg/kg) or PBS was given at the day and one day after every vaccination doses. 15-mer HBx overlapping peptide stimulated IFN-γ ELISpot assay was conducted at day 21 and the result are shown. Statistics: Student's *t*-test. Symbols: * $p<0.05$; ** $p<0.01$.

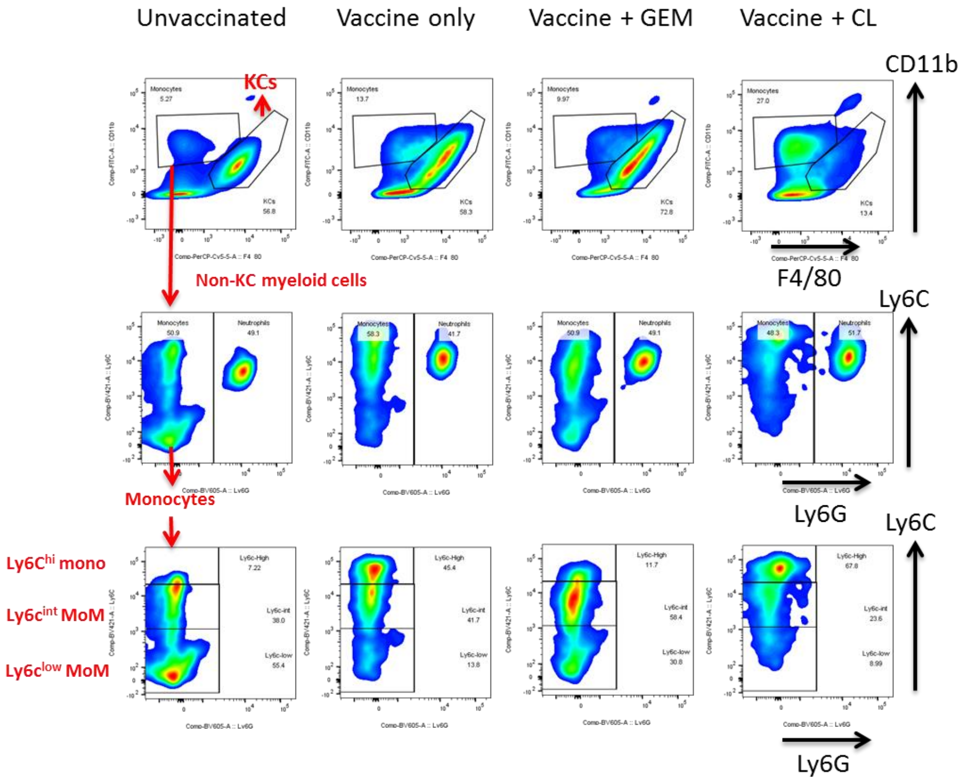

Supplementary figure 8. Representative flow cytometry plots of liver myeloid cell subpopulations after TVGV-HBx vaccination and monocyte depletion

HBV carrier CBA/CaJ mice (N=4) was immunized with TVGV-HBx at day 0. 200μL CL solution was given through intravenous route at day 0. Gemcitabine (40mg/kg) was administrated by intraperitoneal injection at day 0 and 1. Total intrahepatic leukocytes were collected at day 2 post-immunization and subject to flow cytometry analysis. Cells were stained by CD45, CD11b, F4/80, Ly6C and Ly6G. The representative flow cytometry plot for hepatic myeloid cell quantification before and after drug treatment are shown. The gating strategy to classify hepatic myeloid cells subpopulations are illustrated on the unvaccinated lane. KCs, Kupffer cells; Ly6C^{hi} mono, Ly6C^{high} monocytes; Ly6C^{int} MoM, Ly6C-intermediate monocyte derived macrophages; Ly6C^{low} MoM, Ly6C-low monocyte derived macrophages.

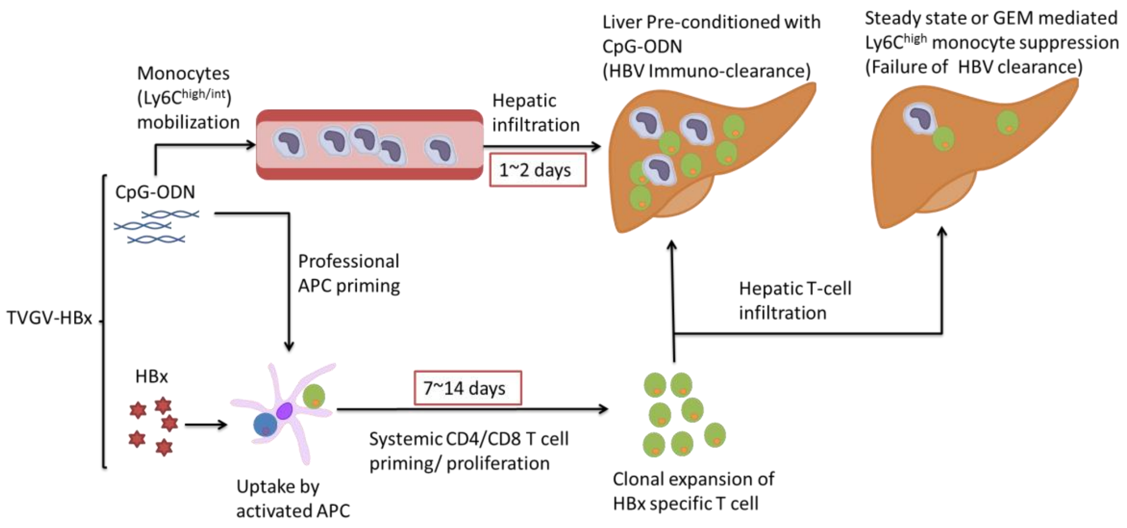

Supplementary figure 9. Schematic diagram of monocyte preconditioning of the liver immune environment and its impact on HBx therapeutic vaccine efficacy

After TVGV-HBx vaccination, the CpG-ODN induces strong monocyte mobilization from bone marrow to the bloodstream and infiltrates the liver within 2 days. At the same time, CpG-ODN also activates professional antigen-presentation cells in the injection site and brings HBx to the secondary lymphoid organs to prime HBx specific CD4 and CD8 T cells. The T cell expansion typically requires 7~14 days after immunization. The activated CD8 T lymphocytes entering the liver exert their HBV clearance function. The monocyte infiltrated liver may provide a favorable environment, including facilitate hepatic T cell proliferation or provide cytokines that benefits T cells to clear HBV expressing cells. Steady state liver or the removal of liver infiltrating Ly6C^{high} monocytes by Gemcitabine (GEM) inhibits the TVGV-HBx immunization mediated HBV clearance.
